# Obituary: in memory of Professor Tadeusz Chojnacki (1932–2020)

**DOI:** 10.1186/s11658-020-00239-4

**Published:** 2020-10-20

**Authors:** Ewa Swiezewska, Anna Chojnacka

**Affiliations:** 1 Department of Lipid Biochemistry, Institute of Biochemistry and Biophysics, Polish Academy of Sciences, Pawinskiego 5A, 02-106 Warsaw, Poland; 2 Department of the History and Theory of Theatre, Institute of Art, Polish Academy of Sciences, Długa 26/28, 00-238 Warsaw, Poland


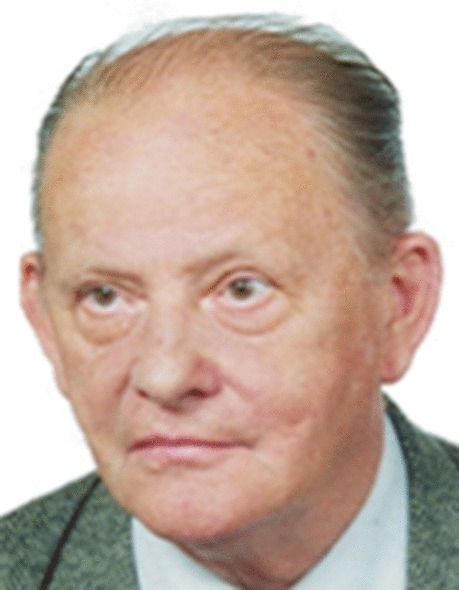
Professor Tadeusz Chojnacki passed away on June 13, 2020.

Professor Chojnacki was a full member of the Polish Academy of Sciences (PAS), a co-founder and vice-director of the Institute of Biochemistry and Biophysics (IBB) of the Polish Academy of Sciences, and a member of the Scientific Council of IBB PAS and the Scientific Councils of a number of scientific institutions.

Professor Chojnacki was also an organizer of the scientific life in Poland, serving as the Secretary and Chairman of the Second Department of Biological Sciences of the Polish Academy of Sciences and the Chairman of the Committee of Biochemistry and Biophysics PAS. He was also among the founders of the journal *Cellular and Molecular Biology Letters* and a member of its Editorial Board.

Professor Chojnacki obtained numerous awards and decorations, including the Commander's Cross of the Order of Polonia Restituta (2008), the Doctor Honoris Causa Award of the Karolinska Institutet in Stockholm (1986), and the Michał Oczapowski Medal (2015).

Professor Chojnacki graduated from the Medical Department of Warsaw Medical Academy in 1955, and a few years after working as a general practitioner he started his professional carrier as a biochemist. From 1958 he was employed at the Institute of Biochemistry and Biophysics PAS in Warsaw, where he received his PhD (1961), headed the Department of Phospholipids/Department of Lipid Biochemistry at IBB PAS (until 2003) and led numerous research projects.

Professor Chojnacki devoted his entire scientific career to lipids.

Initially, Professor Chojnacki’s projects were focused on phospholipid metabolism (PhD dissertation and a postdoctoral project performed with Professor Gordon Ansell, University of Birmingham with two *Nature* papers in 1962 and 1966), nucleotides and glycolipids (collaboration with Professor Tadeusz Korzybski—a doyen of Polish biochemistry), as well as the use of isotope methods in biochemistry and laboratory diagnostics (diagnostics of galactosemia, 1969).

Subsequently, Professor Chojnacki initiated pioneer studies on polyisoprenoids, compounds found in all kingdoms of life, and became a world-recognized authority in this field. Besides studies of polyisoprenoid chemical structure, biosynthesis and biochemistry, he was especially fascinated by chemotaxonomic aspects of plant polyisoprenoids, and their occurrence in various habitats all over the globe. Due to this successfully developed project, IBB PAS became a reference center offering isoprenoid compounds worldwide through the Collection of Polyprenols established by him. Until very recently, Professor Chojnacki remained scientifically active, and the most recent topic which attracted his attention was the use of semisynthetic polyisoprenoid derivatives as lipofectants.

Professor Tadeusz Chojnacki established and effectively conducted numerous collaborative projects with research groups from Poland and internationally. These included projects spanning more than 35 years (1978–2015) together with Professor Gustav Dallner and Professor Kerstin Brismar, University of Stockholm and Karolinska Institutet, resulting in 46 joint publications. Several projects were performed in collaboration with researchers from the Zielinski Institute of Organic Chemistry, Russian Academy of Sciences in Moscow (Professor Vladimir Shibaev, Dr Tatiana Druzhinina and Dr. Leonid Danilov, who incidentally passed away the same day, June 13, 2020), from the Institute of Chemistry, Academy of Sciences of Moldova in Kishinev (Professors Pavel Vlad and Veaceslaw Kulcitki), from Tohoku University in Sendai (Professor Hiroshi Sagami), and many others.

There were also numerous Polish research groups involved in collaboration with Professor Chojnacki, including the Faculty of Biochemistry, Biophysics and Biotechnology Jagiellonian University (Professors Włodzimierz Korohoda, Zbigniew Madeja, Kazimierz Strzałka, Dr. Monika Rak), the Institute of Organic Chemistry PAS (Professors Marek Chmielewski, Jan Pyrek, Włodzimierz Daniewski, Dr. Marek Masnyk), and the Centre of Molecular and Macromolecular Studies PAS in Łódź (Professors Barbara Nawrot and Wojciech Stec). In parallel, Professor Chojnacki’s fascination with plant chemotaxonomy resulted in close collaboration with botanists from numerous botanical gardens and arboreta, including the Botanical Garden—Center for Conservation of Biological Diversity PAS in Powsin (Dr. Andrzej Marczewski, Professors Jerzy Puchalski and Wojciech Dmuchowski), the Arboretum in Trivandrum and in Lucknow, the Arboretum in Bolestraszyce, and the Institute of Dendrology in Kornik.

Professor Chojnacki published approximately 200 papers, many of them considered as landmarks in the field, and he supervised 9 PhD dissertations*.*

Professor Tadeusz Chojnacki was a great scientist, a teacher and mentor who shared his knowledge and skills with us—his students and collaborators.

We will miss his comments on life and science, his advice and suggestions. And we will miss him.

